# From Perception to Metacognition: Auditory and Olfactory Functions in Early Blind, Late Blind, and Sighted Individuals

**DOI:** 10.3389/fpsyg.2016.01450

**Published:** 2016-09-27

**Authors:** Stina Cornell Kärnekull, Artin Arshamian, Mats E. Nilsson, Maria Larsson

**Affiliations:** ^1^Gösta Ekman Laboratory, Department of Psychology, Stockholm UniversityStockholm, Sweden; ^2^Division of Psychology, Department of Clinical Neuroscience, Karolinska InstitutetStockholm, Sweden; ^3^Center for Language Studies and Donders Institute for Brain, Cognition, and Behavior, Radboud UniversityNijmegen, Netherlands

**Keywords:** auditory sense, congenitally blind, compensatory effect, discrimination, episodic odor memory, identification, metacognition, olfaction

## Abstract

Although evidence is mixed, studies have shown that blind individuals perform better than sighted at specific auditory, tactile, and chemosensory tasks. However, few studies have assessed blind and sighted individuals across different sensory modalities in the same study. We tested early blind (*n* = 15), late blind (*n* = 15), and sighted (*n* = 30) participants with analogous olfactory and auditory tests in absolute threshold, discrimination, identification, episodic recognition, and metacognitive ability. Although the multivariate analysis of variance (MANOVA) showed no overall effect of blindness and no interaction with modality, follow-up between-group contrasts indicated a blind-over-sighted advantage in auditory episodic recognition, that was most pronounced in early blind individuals. In contrast to the auditory modality, there was no empirical support for compensatory effects in any of the olfactory tasks. There was no conclusive evidence for group differences in metacognitive ability to predict episodic recognition performance. Taken together, the results showed no evidence of an overall superior performance in blind relative sighted individuals across olfactory and auditory functions, although early blind individuals exceled in episodic auditory recognition memory. This observation may be related to an experience-induced increase in auditory attentional capacity.

## Introduction

Although evidence is mixed, research suggests that blindness may lead to enhanced perceptual and cognitive abilities in the non-visual senses (i.e., compensatory effects). This has been shown for various auditory (Hötting and Röder, 2009), tactile (Occelli et al., 2013), and chemosensory (Kupers and Ptito, 2014) tasks. However, because almost all previous studies have studied one sensory modality at a time, little is known about the generalizability of compensatory effects across sensory modalities and tasks. Moreover, the majority of previous studies have not studied the influence of onset age of blindness on compensatory effects, although several studies indicate that compensatory effects are more pronounced for congenital or early onset blindness than late onset blindness (e.g., Röder and Rösler, 2003; Gougoux et al., 2004; Wan et al., 2010).

Whereas, most evidence suggests that blind and sighted individuals have similar absolute thresholds of hearing and touch (Hötting and Röder, 2009; Occelli et al., 2013; Nilsson and Schenkman, 2016), compensatory effects in blind individuals have been observed for complex and higher-order cognitive tasks (Hötting and Röder, 2009; Frasnelli et al., 2011). For example, studies have shown that congenitally and early blind individuals are better at pitch discrimination and pitch/timbre categorization tasks (Gougoux et al., 2004; Hötting and Röder, 2009; Wan et al., 2010, see also Kupers and Ptito, 2014 for a review). Although Wan et al. (2010) reported higher performance for congenital and early onset blind (but not late onset blind) individuals than sighted for the pitch discrimination and pitch/timbre categorization tasks, there was no evidence that blind individuals had better pitch working memory than the sighted. Therefore, the authors suggested that early onset blindness does not necessarily result in superior auditory functioning in general. Blind individuals have been shown to have better episodic memory for verbal material (Röder et al., 2001) and environmental sounds (Röder and Rösler, 2003) than sighted individuals. Although evidence is scarce regarding compensatory effects in the ability to identify environmental sounds, the only published study investigating this did not find any differences between blind and sighted children (Wakefield et al., 2004).

Considerably less is known about the influence of blindness on olfactory than on auditory abilities, and available evidence is contradictory. For example, whereas some studies have reported no differences between blind and sighted individuals in absolute odor detection thresholds (Kupers et al., 2011; Luers et al., 2014), other work has found that blind individuals exhibit lower (Çomoğlu et al., 2015) or higher (Murphy and Cain, 1986) odor thresholds than the sighted. Furthermore, whereas some studies indicate better odor discrimination ability in blind individuals than in sighted (Cuevas et al., 2009; Çomoğlu et al., 2015), others have reported no performance differences (Smith et al., 1993; Beaulieu-Lefebvre et al., 2011). The empirical evidence favoring the blind over sighted individuals is stronger for semantic olfactory tasks. Blind individuals have been shown to identify more odors than sighted in free identification (Murphy and Cain, 1986; Cuevas et al., 2009; Gagnon et al., 2015). However, a recent study by Sorokowska (2016) reported no effects of blindness across a range of olfactory tasks, including free and cued odor identification. In contrast to research in the auditory sense, olfactory research has not determined whether blindness affects episodic recognition of odors. Possible explanations for the discrepancy of findings reviewed above might be the high diversity in testing procedures (e.g., customized vs. validated olfactory tests) and in study group characteristics across studies (e.g., onset age of blindness, age of participants).

Studying the influence of blindness across different sensory modalities and tasks would add valuable knowledge about the generalizability of compensatory effects. Therefore, we compared compensatory effects between the auditory and olfactory modalities by using a series of analogous tasks that varied in cognitive complexity. To be able to identify potential effects of the onset age of blindness, early blind, late blind, and sighted participants were tested. Participants were tested in absolute threshold, discrimination, identification, episodic recognition, and metacognitive ability (judgments of learning) to predict episodic recognition.

To the best of our knowledge, this is the first study to investigate olfactory episodic recognition and metacognitive abilities in blind individuals. Moreover, the role played by stimulus familiarity, an important factor in episodic memory (Kärnekull et al., 2015), in compensatory effects will be addressed for the first time. The main aims of this study were to examine whether (1) blindness would influence the two sensory modalities and the types of tasks to a similar extent, and if (2) potential compensatory effects would depend on the onset age of blindness.

## Materials and methods

### Participants

Thirty blind (age range: 26–73 years, mean age: 55.5 ± 12.0 years) and thirty sighted individuals (age range: 24–74 years, mean age: 55.2 ± 12.3 years) participated in this study (*n* = 60; 44 females). For each blind participant a sighted sex and age matched participant was recruited (±0–3 years). The blind participants were recruited by advertisements in an audio newspaper and in two newsletters of organizations of the blind and visually impaired, and by contacting participants from previous studies from our laboratory (Nilsson and Schenkman, 2016). The sighted were recruited by advertising at a Swedish website for research volunteers and at notice boards at public places. All participants reported that they had a normal sense of smell and hearing with respect to their age. Two participants were smokers.

Based on the onset age of blindness, the blind participants were divided into two groups: early (*n* = 15) and late blind (*n* = 15). The mean age (±*SD*) and gender distribution for the early blind was 52.5 ± 13.0 years (age range: 26–65, 10 females) and for the late blind 58.5 ± 10.5 years (age range: 44–73, 12 females). The early blind participants were either congenitally blind or had become blind in early childhood (< 2 years old), whereas the majority of the late blind participants became blind in adulthood (one participant in adolescence). Participant characteristics and self-reported causes and onset age of blindness are presented in Table 1.

**Table 1:** **Blind participants' group belonging, age, sex, and self-reported onset age of blindness, cause of blindness, and current visual acuity**.

**No**.	**Group**	**Age (years)**	**Sex**	**Self-reported onset age blindness**	**Self-reported cause of blindness**	**Self-reported visual acuity**
1	Early	65	M	Congenital	Leber's congenital amaurosis	Totally blind
2	Early	57	F	1 year	Retinoblastom	Totally blind
3	Early	53	F	2 years	Retinoblastom	Totally blind
4	Early	52	F	Congenital	Incontinentia pigmenti	Totally blind
5	Early	65	M	2 weeks	Retrolental fibroplasia	Totally blind
6	Early	63	M	Birth	Retrolental fibroplasia	Totally blind
7	Early	58	M	3 months^*^	Fetal infection (undiagnosed)	<0.05
8	Early	63	F	Congenital	Heredo-retinopathia congenitalis	<0.05
9	Early	63	F	Birth	Retrolental fibroplasia	<0.05
10	Early	64	F	Congenital^*^	Glaucoma	<0.05
11	Early	43	F	Congenital^*^	Axenfeld-Rieger syndrome	<0.05
12	Early	45	F	Birth	Retrolental fibroplasia	<0.05
13	Early	26	F	Congenital	Leber's congenital amaurosis	<0.05
14	Early	43	M	Congenital	Retinal degeneration	<0.05
15	Early	28	F	Congenital	Leber's congenital amaurosis	<0.05
16	Late	48	M	40 years	Retinis pigmentosa	<0.05
17	Late	56	F	46 years	Retinis pigmentosa	Totally blind
18	Late	56	F	20 years	Glaucoma	Totally blind
19	Late	73	F	62 years	Undetermined	<0.05
20	Late	67	M	57 years	Retinis pigmentosa	<0.05
21	Late	61	F	58 years	Keratitis	Totally blind
22	Late	58	F	38 years	Retinis pigmentosa	Totally blind
23	Late	70	F	69 years	Diabetic retinopathy	<0.05
24	Late	53	F	51 years	Cataract, impaired cornea	<0.05
25	Late	45	F	29 years	Tumors pressing on the optic nerve	Totally blind
26	Late	45	F	20 years	Stargardt's disease	<0.05
27	Late	56	M	15 years	Retinal detachment	Totally blind
28	Late	44	F	39 years	Tumors pressing on the optic nerve	<0.05
29	Late	73	F	50 years	Macular degeneration	<0.05
30	Late	72	F	28 years	Optic nerve inflammation	<0.05

* *Specific participants reported they were born with visual acuity of < 0.1 (legally blind)*.

The study was approved by the Regional Ethical Review Board in Stockholm (2015/369-31/4), and all participants provided written informed consent before the study. The participants were compensated for participating in the study (voucher à 600 SEK) and travel expenses were reimbursed.

### Materials and procedure

After being orally informed about the general aim and the procedure of the study the participant provided written informed consent. The study comprised of an olfactory session in a custom-made olfactory testing room with high-pressure ventilation and an auditory session in a custom-made and sound-isolated auditory testing room. The two test sessions were separated with a 30-min pause. The sensory modality order was randomized across matched pairs of blind and sighted participants. The olfactory and auditory sessions consisted of an absolute threshold test, a discrimination test, an identification test, and an episodic recognition test, respectively. After the encoding of stimuli in the recognition test, a global judgment of learning (JOL) was made. Lastly, a questionnaire targeting demographic and health information, volitional imagery ability for odors and sounds, and attention to odors was answered. Participants' imagery ability for odors (VOIQ; Gilbert et al., 1998) and sounds (CAIS; Willander and Baraldi, 2010), and attention to odors (Wrzesniewski et al., 1999; Stevenson and Case, 2004) are presented in the Supplementary Material. Information about the study was given orally and the responses were registered by the experimenter. All participants were blindfolded at testing.

#### Absolute odor threshold

A single staircase detection threshold method for n-butanol with a three-alternative forced choice was applied with the Sniffin' Sticks olfactory test (Hummel et al., 1997). The test comprises sixteen n-butanol dilutions (1–16). At each dilution level, three odor pens are presented in a random order, of which one contains the n-butanol dilution and the other two contain the solvent (deionized aqua conservata). The task is to identify the pen that smells different from the other two, that is, the pen containing the n-butanol dilution, with a three-alternative forced choice procedure. First, and to certify that the participant was not anosmic, the triplet representing the lowest n-butanol dilution (i.e., level 1) was presented. Then, the threshold test was initiated at dilution level 12 with decreasing levels by taking two steps at a time (12, 10, 8, etc.) until the participant made two correct responses at the same concentration level. Please note that the common procedure is to start at level 15 or 16, but because of the relatively old ages in this study sample we started at level 12. When two correct responses were made in a row a cross was marked in the protocol and the staircase was reversed, that is, pens at a higher dilution step were presented. An incorrect response led to odor presentations at a lower dilution step. The individual threshold was defined as the mean of the last four of seven staircase reversals.

#### Odor quality discrimination

The Sniffin' Sticks odor discrimination test (Hummel et al., 1997) was used for assessing odor quality discrimination ability. The test comprises 16 triplets of odor pens with varying smells. Each triplet contains two identical odors and one that differs from the other two. With a three-alternative forced choice procedure the task is to identify the pen that smells different. Each odor was smelled once and for ~3 s. The triplets were presented with an inter-stimulus interval of ~25 s and the presentation order was individually randomized across the matched participants. Discrimination performance was defined as the number of correct responses (maximum = 16).

#### Episodic odor recognition and identification

A total of 24 odors were used, of which half was high familiar and half was low familiar. The high familiar odors (*n* = 12) were selected from the Sniffin' Sticks identification tests whereas the selection of low familiar odors (*n* = 12) was based on pilot studies (see list of odors in Table 2). The low familiar odors were prepared in empty Sniffin' Sticks in our lab and where extremely hard to name, as these stimuli only had chemical or brand names. The participants rated familiarity on a 7-point scale (1 = *not familiar at all*, 7 = *very familiar*) and as expected, the high familiar odors were perceived as more familiar (*M* = 5.26, *SD* = 0.82) than the low familiar odors (*M* = 3.17, *SD* = 0.79). Half of the odors (6 high familiar, 6 low familiar) served as targets to be remembered for a subsequent memory test and the other half (6 high familiar, 6 low familiar) served as distractors at the memory test. The presentation order of the odors was individually randomized across the matched participants.

**Table 2:** **Odor sets of high and low familiarity**.

**High familiar odors**	**Low familiar odors**
Banana^a^	Ethyl-diethylmalonate^b^
Caramel^a^	Glutaraldehyde^b^
Fish^a^	Heptanal^b^
Garlic^a^	1-Hexanol^b^
Grass^a^	Hexanoic acid^b^
Lavender^a^	Isobuthyl quinoline^c^
Lilac^a^	Isobuthyl salicylate^b^
Liquorice^a^	Lemorosa^c^
Mushroom^a^	o-Toluidine^b^
Orange^a^	Styryl acetate^c^
Peach^a^	Violet leaf^c^
Peppermint^a^	9-Decen-1-ol

a *Selected from Sniffin' Sticks Identification tests*.

b *Donated by the Department of Organic Chemistry at Stockholm University*.

c *International Flavors and Fragrances Inc*.

At encoding, the participant was instructed to smell 12 target odors (6 high familiar, 6 low familiar) and to remember as many as possible for a subsequent memory test. Each odor was smelled once and for ~3 s. To minimize potential effects of adaptation there was an inter-stimulus interval of ~25 s. Immediately after the encoding of odors, the participant made a global JOL (Koriat et al., 2004), by estimating the percentage of odors that would be remembered in the subsequent recognition test. During the retention interval, which lasted for ~8–9 min, a verbal fluency task was administered using the verbal associative fluency test (FAS, Spreen and Benton, 1969; Ross et al., 2006) or a modified version with the letters R, E, and P (see Ross et al., 2006 for a similar paradigm) to minimize potential effects of verbal rehearsal of the presented odors.

At the episodic recognition test, a total of 24 odors were presented, consisting of all target odors intermixed with the same number of distractor odors (6 high familiar, 6 low familiar). For each odor, the task was to decide whether it had been presented previously or not (yes/no) using a 2- alternative forced choice procedure (cf. Croy et al., 2015; Kärnekull et al., 2015). Following each recognition response, participants rated the odor with regard to perceived pleasantness on a 7-point scale (1 = *not pleasant at all*, 7 = *very pleasant*) and perceived familiarity on a 7-point scale (1 = *not familiar at all*, 7 = *very familiar*). Lastly, the participant was asked to identify the odor. The identification responses were dichotomously scored (1 = correct, 0 = incorrect), and the total number of correct responses was calculated. Identification performance was analyzed for the high familiar odors only (maximum = 12).

#### Absolute auditory threshold

The hearing of all participants was tested using an audiometer (Interacoustic Diagnostic Audiometer, model AD226). As a measure of absolute auditory threshold, the pure-tone average thresholds (PTAs) were calculated across the left and right ears for the frequencies 0.5, 1, 2, and 4 kHz (Schenkman and Nilsson, 2010).

#### Timbre discrimination

To mimic the olfactory discrimination task, which involves discrimination of perceived character of odors, we developed an analogous auditory task based on discrimination of perceived character (or timbre) of sounds. In this task, the participants discriminated between complex tones with slightly different harmonic-amplitude pattern. Perceptually, the tones differed in timbre, but were all of equal loudness and pitch. The complex tones were created by adding twelve zero-phase sinusoids in a harmonic series with fundamental frequency, f_0_ = 350 Hz (f_1_ = 700, f_2_ = 1050, …, f_11_ = 3850 Hz). The amplitudes of the eleven harmonics f_1_, f_2_,…, f_11_ were randomly selected (without replacement) from the set of eleven integer levels between −11 and −1 dB re f_0_ = 0 dB.

A pair of to-be-discriminated tones was created by switching the amplitudes of harmonics f_2_ and f_4_. For example, if a complex tone had this pattern of amplitudes: < 0, −4, −**9**, −1, −**6**, −11, −8, −10, −7, −2, −5, −3 dB> for f_0_ through f_11_, then the comparison tone would have this pattern: < 0, −4, −**6**, −1, −**9**, −11, −8, −10, −7, −2, −5, −3 dB>, (difference in bold). We created a large set of such pairs and tested these in a pilot study. From the result we choose 32 pairs for the main experiment that were not too easy and not impossible to discriminate.

In the experiment, the complex tones of a pair were presented in three successive intervals, one member of the pair was presented once and the other twice in a random order (inter-stimulus-interval = 200 ms). The participant's task was to decide which of the intervals that was different. The presentation order of the trials was randomized across the matched pairs of blind and sighted participants. Discrimination performance was defined as the number of correct responses (maximum = 32).

Each complex tone lasted for 400 ms, including 30 ms fade-in and out (cosine ramp). The tones were presented diotically in earphones at an overall sound pressure level (SPL) of 80 dBA.

#### Episodic sound recognition and identification

A total of 60 environmental sounds were used, half of which were considered as high familiar and half as low familiar based on pilot studies (see list of sounds in Table 3). The sounds were selected from a large sound database on CDs (BBC Sound Effects Library-Original Series, UK) and from an online collaborative sound database (Freesound, www.freesound.org). The sounds were edited into a duration of 2–3 s and converted into stereo (if not already so) by using a sound editor and recorder program [Audacity(R)]. As expected, the participants rated the high familiar sounds (*M* = 5.35, *SD* = 0.86) as more familiar than the low familiar sounds (*M* = 3.73, *SD* = 0.98), on a 7-point scale (1 = *not familiar at all*, 7 = *very familiar*). Half of the sounds (15 high familiar, 15 low familiar) served as targets to be remembered for a subsequent memory test and the other half (15 high familiar, 15 low familiar) served as distractors at the memory test. A unique random presentation order was used for each pair of blind and matched sighted participant. The stereo recordings were presented in earphones at an overall SPL ranging from 54 to 80 dBA in the ear with the higher SPL.

**Table 3:** **Sound sets of high and low familiarity**.

**High familiar sounds**	**Low familiar sounds**
Seawash	Wood fire
Clock ticking	Golf bunker shot
Turning book pages	Skiers passing
Tractor started	Electric kettle
Table tennis	Linoleum floor squeaks
Windshield wipers	Rain on pots
Pulling a pint	Shaving cream
Roulette wheel	Cows walking past
Bread being sliced	Burners
Hair dryer	Fry egg
Footsteps in snow	Seatbelt released
Horse trot	Cattle in hay
Footsteps in shingle	Gambling chip sorting machine
Car started	Donkey walking past
Inflating rubber dingy	Printing machinery
Stoking boiler	Fencing practice
Sail flapping	Bottle cleaning industry
Car indicators	Bilge pump
Electronic drill	Pumping water by hand
Bath emptied	Ice skating spin
Bath room fan	Bicycle ride
Car electric windows	Blinds up and down
Eating a cracker	Can opener
Lighter	Peeling an orange
Paper rip	Ice cube tray
Pieces of glass	Stapler
Scissors	Squeeze a lemon
Velcro	Pencil erasing
Zipper	Peeling apples
Cards shuffling	Buttering a toast

The participant was instructed to listen to 30 target sounds (15 high familiar, 15 low familiar) and to remember as many as possible for a subsequent memory test. Immediately after the encoding of sounds, the participant made a global JOL (Koriat et al., 2004), by estimating the percentage of sounds that would be remembered for the subsequent recognition test. During the retention interval, which lasted for ~8–9 min, a verbal fluency task was conducted (analogous to the one used for the odor recognition test, Section Episodic Odor Recognition and Identification).

The procedure for the episodic recognition test was identical to the one used for odors. At the episodic recognition test a total of 60 sounds were presented, consisting of all target sounds intermixed with the same number of distractor sounds (15 high familiar, 15 low familiar). For each sound, the task was to decide whether it had been presented previously or not (yes/no) with a two-alternative forced choice procedure. This was followed by a rating of the perceived pleasantness on a 7-point scale (1 = *not pleasant at all*, 7 = *very pleasant*) and perceived familiarity on a 7-point scale (1 = *not familiar at all*, 7 = *very familiar*). Lastly, the participant was asked to identify the sound. The responses were dichotomously scored (1 = correct, 0 = incorrect) and a total number of correct responses was calculated for each set of sounds (maximum = 30).

#### Sound equipment

The sounds were presented using a custom-build computer (OS: Microsoft Windows 7), connected to a soundcard (RMEHDSPe FX), D/A converter (RME ADI-8 QS), earphone amplifier (LP Phone-amp G109), and earphones (Beyerdynamic DT 990 Pro). The sounds were created or reproduced from files using Python 2.7 and the package PsychoPy (Peirce, 2007).

### Data analyses

A multivariate analysis of variance (MANOVA) was conducted on the olfactory and auditory tests with group (early blind, late blind, sighted) as the between-subjects factor and modality (olfactory, auditory) as the within-subjects factor. Before conducting the analysis the data were transformed into *z*-scores. The MANOVA was followed-up with simple contrasts of mean group differences for each of the tasks (in total 24 independent-samples *t*-tests, three group comparisons for each of the eight tasks). The Bonferroni-test was used to correct for multiple comparisons to keep the family-wise type 1 error rate at 0.05. Note that it is a conservative correction, where alpha for each contrast is ~0.002 (0.05/24). *d'* served as an index of episodic recognition performance and is an unbiased measure of sensitivity (Macmillan and Creelman, 2005). In the signal detection theory model, *d'* is defined as the *z*-transformed difference between proportions of hits (H) and false alarms (FA); [*d'* = *z* (H)–*z* (FA)] (Macmillan and Creelman, 2005). Hit and false alarm rates of 1 and 0 were adjusted to 1—1/(2N) and 1/(2N), respectively (Macmillan and Creelman, 2005). Hit and false alarm rates and response bias (c) for odors and sounds are presented in the Supplementary Material (Figures S1, S2).

Furthermore, because there was no interaction effect between group (early blind, late blind, sighted) and stimulus familiarity (low familiar, high familiar) on recognition performance for neither odors nor sounds this factor was collapsed in the analyses presented below (but see Supplementary Material, Table S1).

As noted above, odor identification performance was analyzed for high familiar odors only, as only these stimuli had corresponding names. For consistency, sound identification performance is also presented for high familiar sounds only, and this is further justified by the observation that there was no interaction effect of group and stimulus familiarity on sound identification (Supplementary Material, Table S1 and Figure S3).

Also, correlations between judgments of learning (JOLs) and episodic recognition (*d'*) as a function of group were computed. The analyses were conducted in SPSS and R (R Core Team, 2014).

## Results

### Multivariate analysis

**Figure 1:**
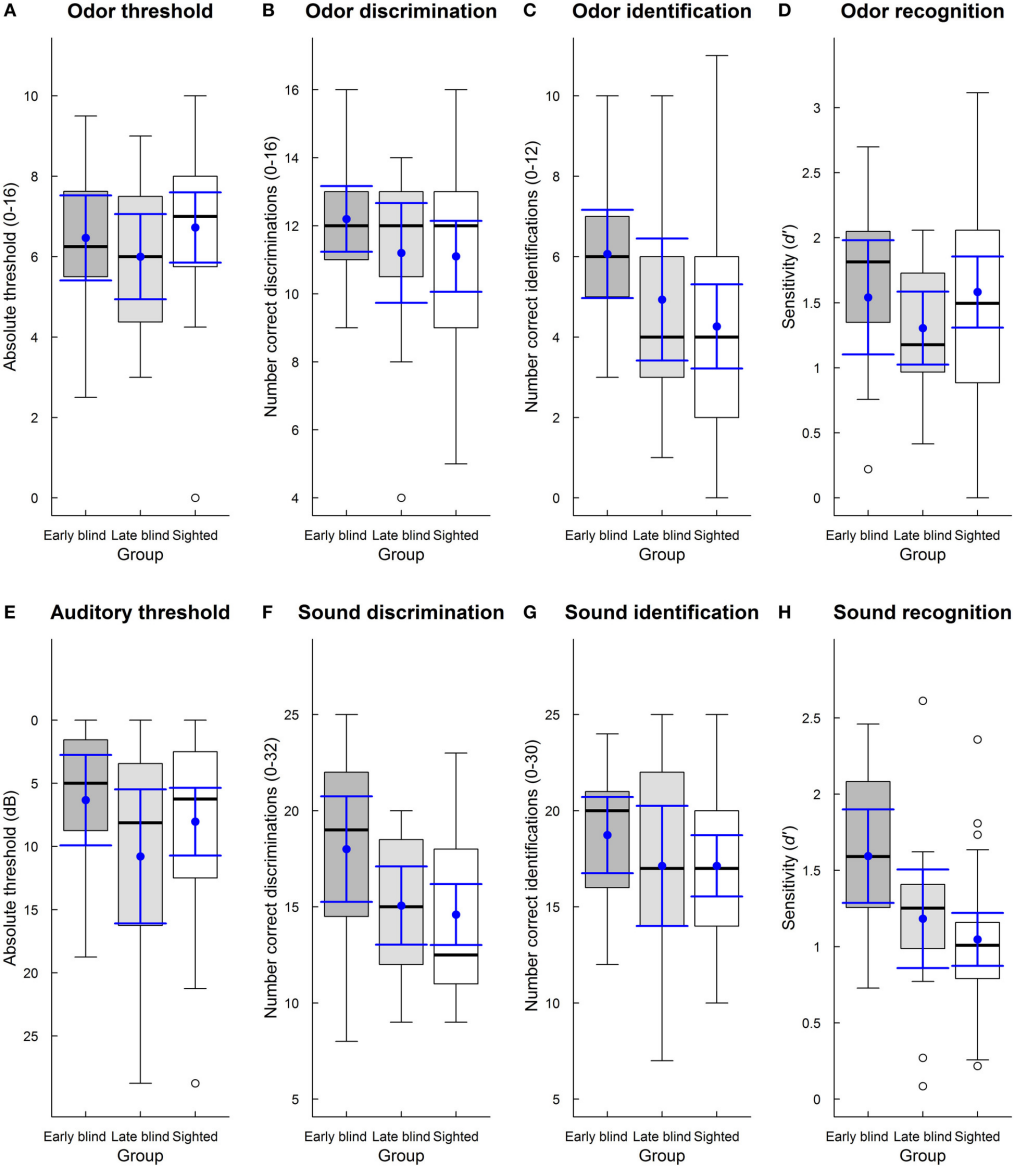
**(A–H)** Boxplots of absolute odor threshold **(A)**, odor discrimination **(B)**, odor identification of high familiar stimuli **(C)**, episodic odor recognition (*d*') **(D)**, absolute auditory threshold **(E)**, sound discrimination **(F)**, sound identification of high familiar stimuli **(G)**, and episodic sound recognition (*d*') **(H)**. Boxplots are displayed separately for early blind (dark gray boxes), late blind (light gray boxes), and sighted (white boxes) participants. The boxes indicate the 25th, 50th (median), and 75th percentiles of the distribution (lower, middle, and upper horizontal lines of the box). The upper hinges indicate the maximum value of the variable located within a distance of 1.5 times the inter-quartile range above the 75th percentile. The lower hinges indicate the corresponding distance to the 25th percentile value. Circles indicate values outside these hinges (outliers). The means and 95% confidence intervals (dots and error bars in blue) are superimposed on the boxplots.

The MANOVA on the olfactory and auditory tests (threshold, discrimination, identification, and episodic recognition) showed no significant effect of group [Wilk's λ = 0.82, *F*_(8, 108)_ = 1.37, *p* = 0.22], modality [Wilk's λ = 0.98, *F*_(4, 54)_ = 0.30, *p* = 0.87], or interaction between group and modality [Wilk's λ = 0.79, *F*_(8, 108)_ = 1.71, *p* = 0.10]. However, visual inspection of group data (Figure 1) did not suggest a random pattern of group differences: for some tasks, blind performed better than sighted, and early blind performed better than late blind, consistent with what we would expect from previous research. We therefore proceeded by calculating between-group contrasts for each task using independent-samples *t*-tests (to control for multiple comparison, the *p*-values reported below should be evaluated at a Bonferroni corrected alpha = 0.05/24 = 0.002).

### Olfactory tests

Early blind (*M* = 6.47, *SD* = 1.91), late blind (*M* = 6.00, *SD* = 1.92), and sighted participants (*M* = 6.73, *SD* = 2.34) had similar absolute odor thresholds (Figure 1A; independent-samples *t*-tests, uncorrected *p*s > 0.05 to be evaluated against Bonferroni corrected α = 0.05/24 = 0.002).

For odor discrimination ability, there were relatively modest differences between early blind (*M* = 12.20, *SD* = 1.74), late blind (*M* = 11.20, *SD* = 2.65), and sighted participants (*M* = 11.10, *SD* = 2.80; Figure 1B; *p*s > 0.05).

There was a similar pattern of findings for identification of high familiar odors, where early blind participants (*M* = 6.07, *SD* = 1.98) performed slightly better than late blind (*M* = 4.93, *SD* = 2.74) and sighted participants (*M* = 4.27, *SD* = 2.80; Figure 1C; *p*s > 0.03).

Further, as shown in Figure 1D, early blind (*M* = 1.54, *SD* = 0.79), late blind (*M* = 1.31, *SD* = 0.51), and sighted participants (*M* = 1.58, *SD* = 0.73) had similar episodic odor recognition performances as indexed by *d'*, with mean differences corresponding to < 1 recognized item (from a total of 24; *p*s > 0.05).

### Auditory tests

Early blind (*M* = 6.33, *SD* = 6.47), late blind (*M* = 10.79, *SD* = 9.58), and sighted participants (*M* = 8.04, *SD* = 7.18) had similar absolute auditory thresholds (Figure 1E; *p*s > 0.05).

As shown in Figure 1F, early blind participants (*M* = 18.00, *SD* = 4.96) correctly discriminated more sounds than late blind (*M* = 15.07, *SD* = 3.67) and sighted participants (*M* = 14.60, *SD* = 4.25; *p*s > 0.02), although the differences did not reach statistical significance.

The group differences in identification ability of high familiar sounds were relatively small, with early blind (*M* = 18.73, *SD* = 3.58) performing slightly better than late blind (*M* = 17.13, *SD* = 5.64) and sighted participants (*M* = 17.13, *SD* = 4.27; Figure 1G; *p*s > 0.05).

As shown in Figure 1H, episodic sound recognition (*d'*) was better for early blind (*M* = 1.59, *SD* = 0.55) than late blind (*M* = 1.18, *SD* = 0.58) and sighted participants (*M* = 1.05, *SD* = 0.46). The mean difference between early blind and sighted participants was substantial (Cohen's *d* = 1.10) and corresponded to five recognized items (from a total of 60; *p* = 0.001). The differences between early blind and late blind participants and between late blind and sighted participants, however, were smaller (*p*s > 0.05).

### Relationships of judgments of learning (JOLs) with episodic odor and sound recognition

**Figure 2:**
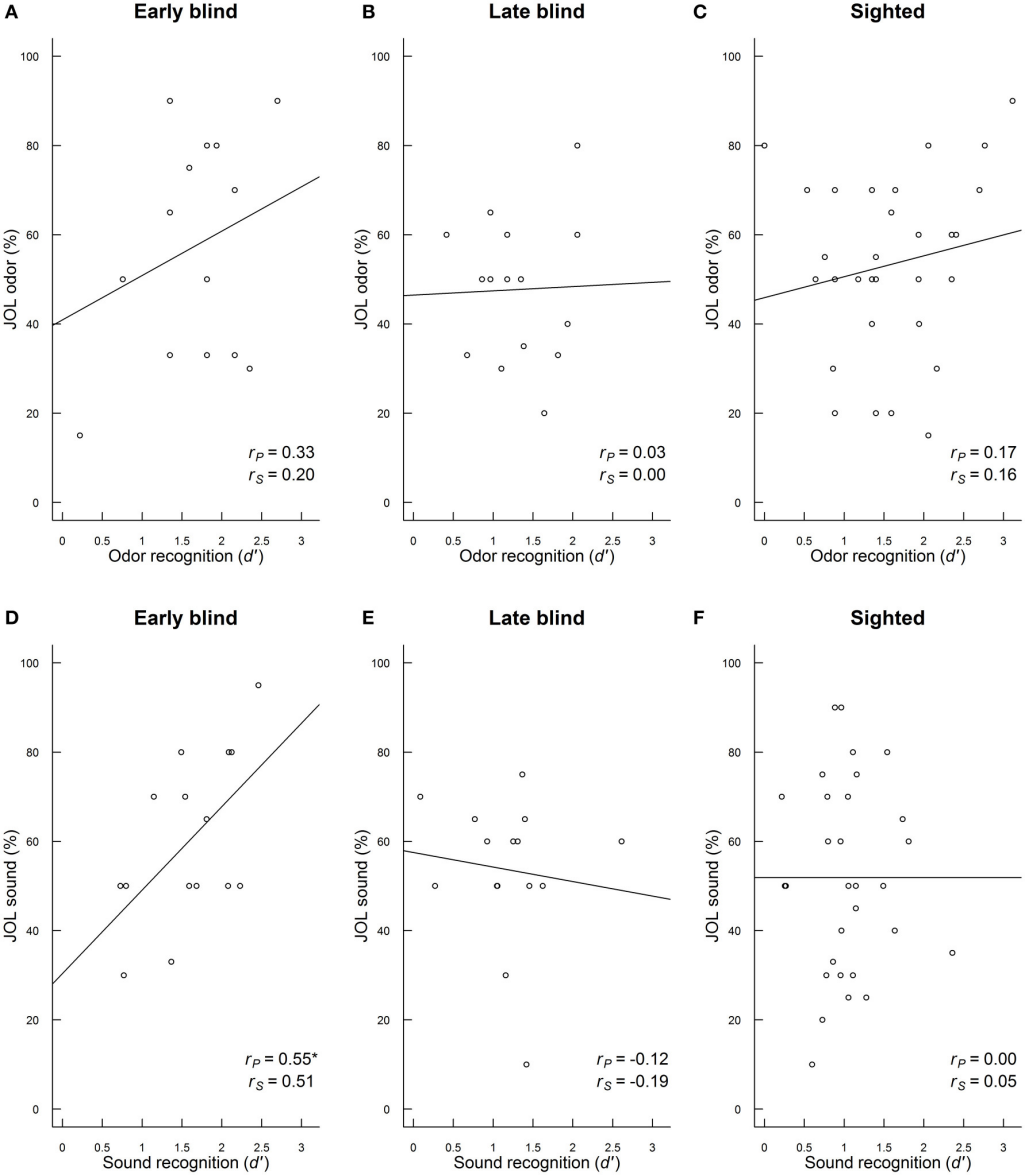
**(A–F)** Correlations of judgments of learning (JOLs) with episodic recognition (d') for odors **(A–C)** and sounds **(D–F)** in early blind, late blind, and sighted participants. The Pearson product-moment correlation coefficient (rP) with fitted regression line (ordinary least squares), and the Spearman's rank correlation coefficient (rS) are depicted. ^*^p < 0.05.

Participants' metacognitive skills for predicting episodic recognition performance were examined by analyzing the relationship between judgments of learning (JOLs) and episodic recognition performance (*d'*) in early blind, late blind, and sighted participants for odors (Figures 2A–C) and sounds (Figures 2D–F), respectively. The positive relationships between JOLs and recognition (*d'*) were stronger for the early blind participants than for late blind and sighted participants and were more pronounced for sound recognition. It should be noted, however, that neither of the differences between groups reached statistical significance. The pairwise comparisons were *z* tested according to Fisher's formula, after each Pearson correlation coefficient had been converted into *r*'. For olfaction, there were no significant differences between early blind and late blind (*z* = 0.77), early blind and sighted (*z* = 0.49), or late blind and sighted participants (*z* = −0.41; *p*s > 0.05). Likewise, no significant differences were found between early and late blind (*z* = 1.81), early blind and sighted (*z* = 1.78), or late blind and sighted participants (*z* = −0.35; *p*s > 0.05).

## Discussion

The present study found that there were no overall effect of blindness on the olfactory and auditory functions and no interaction between the modalities. However, a closer inspection of the data with between-group contrasts showed that early blind participants were better at auditory episodic recognition than the sighted controls. In contrast to the auditory modality, there was no empirical support for group differences in any of the olfactory tasks.

In line with previous research (e.g., Luers et al., 2014; Nilsson and Schenkman, 2016), no effects of blindness were found for auditory or olfactory absolute thresholds. In contrast, previous studies have found blind-over-sighted advantages in pitch discrimination and pitch/timbre categorization tasks (Gougoux et al., 2004; Wan et al., 2010). Regarding olfactory discrimination, evidence is more mixed (e.g., Beaulieu-Lefebvre et al., 2011; Luers et al., 2014; Çomoğlu et al., 2015). The present study showed that early blind participants had nominally better timbre and odor discrimination than the late blind and sighted, although these differences proved not reliable.

Little is known about blind individuals' identification ability of environmental sounds. Previous research has not found any differences between blind and sighted children (Wakefield et al., 2004). This present study did not find any substantial group effects for either sound or odor identification, although early blind participants were slightly better at the tasks than the late blind and sighted (e.g., Cuevas et al., 2009; Gagnon et al., 2015). As noted, evidence is also scarce with regard to episodic memory of auditory information in blind and sighted individuals, although some evidence suggests that blind people have better memory than sighted for verbal material read aloud (Röder et al., 2001; Raz, 2004; Hötting and Röder, 2009). In a similar vein, Röder and Rösler (2003) tested episodic recognition of high familiar environmental sounds and found that congenitally blind participants had better memory than the sighted, although the late blind did not differ significantly from either early blind or sighted participants (cf. Cobb et al., 1979). This finding was extended in the present work by showing that auditory episodic recognition was better in early blind participants than late blind and sighted participants, irrespective of sound familiarity. This outcome suggests that group differences might not only be due to general training effects of high familiar environmental sounds. The observation that early blind, but not late blind, participants performed better than the sighted suggests that compensatory effects may be dependent on the onset age of blindness. In contrast to the group differences found for episodic sound recognition, no differences were observed in odor memory. Hence, blind individuals' documented superior episodic memory for various types of auditory information (see Hötting and Röder, 2009 for a review), does not appear to generalize to olfactory information.

There were no statistically significant group differences in metacognitive abilities, but the correlation between JOL and memory performance was stronger for early blind participants than for late blind and sighted participants, especially for memory of sounds.

Taken together, we found a compensatory effect in early blind individuals for auditory episodic recognition but not any substantial differences between the groups for any of the olfactory tasks. The findings suggest that there is likely no general compensatory effect across auditory or olfactory functions in general, but that specific auditory abilities, such as memory for sounds, may benefit from blindness. Next to vision, the auditory sense is the most important sense for spatial navigation, which makes it crucial for everyday functioning in blind individuals. Research has also shown that blind people may develop enhanced skills for this purpose (e.g., sound localization in the periphery: Fieger et al., 2006; echolocalization: Dufour et al., 2005; Schenkman and Nilsson, 2010), although it should be noted that blind individuals have been shown to perform worse than sighted in specific spatial tasks (e.g., Lewald, 2002, see Hötting and Röder, 2009 for a review). Compared to the auditory sense, olfaction appears to be less important in everyday life (Keller and Malaspina, 2013), and consequently, blind participants may attend to ambient odors less than to surrounding sounds. These circumstances might explain why no compensatory effects were observed for olfactory functions in this study. There are several possible reasons for why early blind participants had better auditory memory performance than the sighted. For example, training and increased selective attention toward non-visual information and decreased proneness to interference from task-irrelevant stimuli have been put forward as candidate factors that may drive a sharpening of the non-visual skills in blind people (e.g., Hötting and Röder, 2004, 2009; Collignon et al., 2006; Pigeon and Marin-Lamellet, 2015), although the precise underlying mechanisms still remain to be elucidated (Occelli et al., 2013). Another potentially important factor is cross-modal brain plasticity of the occipital cortex in blind individuals (Pascual-Leone et al., 2005; Voss et al., 2008; Kupers et al., 2011). In the present study, the most pronounced performance difference in auditory episodic recognition was observed between the early blind and sighted participants. This is in line with earlier work where blindness onset age has also proved crucial (Hötting and Röder, 2004; Röder et al., 2004; Occelli et al., 2013).

The main limitation of this study is the relatively low statistical power, due to a limited sample size. This is, unfortunately, a common problem when studying blind individuals. The findings should be replicated before any definite conclusions can be made. Although a recent larger scale study also reported no effects of blindness in odor thresholds, discrimination, or identification (Sorokowska, 2016), we cannot rule out that certain differences exist in the population. The issue of power was also clear with regard to group differences in metacognitive abilities. Since this is, to the best of our knowledge, the first time metacognition was assessed in blind individuals it calls for further investigation. Moreover, future studies should not only assess judgments of learning globally but also as item-by-item (Koriat, 1997). Such a procedure would provide a more precise measure of metacognitive ability and at the same time enable evaluation of its potential relationship with item familiarity. Finally, although this study did not find any compensatory effects in memory for either low or high familiar odors, future studies should investigate whether this finding also applies to a broader set of ecologically relevant odors (e.g., smell of burnt or gas leak).

In conclusion, early blind individuals showed a higher performance in episodic recognition of environmental sounds, suggesting that age at blindness onset may be an important factor for compensatory processes to occur. No evidence of superior performance in the other auditory or olfactory functions in blind individuals was observed.

## Author contributions

AA and ML developed the study concept. SC, AA, MN, and ML jointly designed the study. SC produced the olfactory and auditory testing protocols. MN programmed the auditory tests. SC tested the participants and analyzed the data together with AA, MN, and ML. AA and SC drafted the manuscript. ML and MN revised the manuscript.

## Funding

This work was funded by a program grant entitled “Our unique sense of smell” awarded by the Swedish Foundation for Humanities and Social Sciences (M14-0375:1) to ML. This work was also supported by funds to MN from Promobilia (14046) and to AA from the International Postdoc grant from the Swedish Research Council.

### Conflict of interest statement

The authors declare that the research was conducted in the absence of any commercial or financial relationships that could be construed as a potential conflict of interest.
