# Supplementary material for: From Perception to Metacognition: Auditory and Olfactory Functions in Early Blind, Late Blind, and Sighted Individuals
Source: Front Psychol. 2016 Sep 27;7:1450. doi: 10.3389/fpsyg.2016.01450 (PMC5037222; doi:10.3389/fpsyg.2016.01450)
Supplement: Supplementary file 1 [file Table1.pdf]

Supplementary Material

From perception to metacognition: Auditory and olfactory functions in early blind, late blind, and sighted individuals

Stina Cornell Kärnekull[1†], Artin Arshamian[1,2,3†*] Mats E Nilsson[1], Maria Larsson[1]
*** Correspondence:** Dr. Artin Arshamian: artin.arshamian@ki.se

Table S1. Results from separate univariate analyses of variance (ANOVAs) on hit rate, false alarm rate, sensitivity (*d'*), and response bias (*c*) with familiarity (high, low) and group (early blind, late blind, sighted) as independent variables are presented for odors and sounds, respectively. In addition, the results from an ANOVA on sound identification with familiarity (high, low) and group (early blind, late blind, sighted) as independent variables are presented.

Modality	Dependent variable	Independent variable	*F*	df
Odor	Hit rate	Group	0.54	2,57
		Familiarity	22.28^{***}	1,57
		Group * Familiarity	1.56	2,57
	False alarm rate	Group	1.00	2,57
		Familiarity	3.13	1,57
		Group * Familiarity	0.69	2,57
	Sensitivity (*d'*)	Group	0.64	2,57
		Familiarity	18.69^{***}	1,57
		Group * Familiarity	1.78	2,57
	Response bias (*c*)	Group	0.89	2,57
		Familiarity	3.65	1,57
		Group * Familiarity	0.30	2,57
Sound	Hit rate	Group	1.12	2,57
		Familiarity	37.79^{***}	1,57
		Group * Familiarity	0.54	2,57
	False alarm rate	Group	2.84	2,57
		Familiarity	0.26	1,57
		Group * Familiarity	1.52	2,57
	Identification	Group	1.13	2,57
		Familiarity	796.14^{***}	1,57
		Group * Familiarity	0.12	2,57
	Sensitivity (*d'*)	Group	6.11^{**}	2,57
		Familiarity	13.35^{**}	1,57
		Group * Familiarity	0.55	2,57
	Response bias (*c*)	Group	0.35	2,57
		Familiarity	24.64^{***}	1,57
		Group * Familiarity	1.57	2,57

Note. $^{**}p < 0.01$, $^{***}p < 0.001$
