# Supplementary material for: From Perception to Metacognition: Auditory and Olfactory Functions in Early Blind, Late Blind, and Sighted Individuals
Source: Front Psychol. 2016 Sep 27;7:1450. doi: 10.3389/fpsyg.2016.01450 (PMC5037222; doi:10.3389/fpsyg.2016.01450)
Supplement: Supplementary file 2 [file DataSheet1.pdf]

Supplementary Material

From perception to metacognition: Auditory and olfactory functions in early blind, late blind, and sighted individuals

Stina Cornell Kärnekull[1†], Artin Arshamian[1,2,3†*] Mats E Nilsson[1], Maria Larsson[1]
*** Correspondence:** Dr. Artin Arshamian: artin.arshamian@ki.se

1 Attention to odors and imagery ability for odors and sounds

1.1 Method

Attention to odors was assessed by using the following three items from a large questionnaire regarding sensory experiences (Stevenson & Case, 2004, see also Wrzesniewski, McCauley, & Rozin, 1999): "How would you rate the amount of attention you pay to odors relative to other people your age?" (1 = *A lot less*, 2 = *A little less*, 3 = *About the same*, 4 = *A little more*, 5 = *A lot more*), "Do you think you could identify someone important to you on the basis of their smell?" (*No, Unsure, Yes*), and "Yesterday how many smells (if any) do you remember noticing?".

Imagery ability was assessed by using the Vividness of olfactory imagery questionnaire (VOIQ**;** Gilbert, Crouch, & Kemp, 1998), translated into Swedish, and the Clarity of auditory imagery scale (CAIS; Willander & Baraldi, 2010). The VOIQ consists of sixteen items where the participant is asked to mentally imagine the odor of an object or activity, and rate its vividness on a 5-point scale (1 = *no odor at all, you only 'know' you are thinking of an odor*, 2 = *Vague and dim*, 3 = *Moderately realistic and vivid*, 4 = *Realistic and reasonably vivid*, 5 = *perfectly realistic and as vivid as the actual odor*). Compared to the original scale, this scale is reversed. The CAIS consists of 16 items where the participant is asked to mentally imagine the sound of an object or activity, and rate its clarity on a 5-point scale with verbal labels on the end points (1 = *very clear*, 5 = *not at all*).

1.2 Results

The ratings of the amount of attention to odors was higher for late blind participants (M = 4.47, SD = 0.74) than for early blind (M = 3.73, SD = 0.80) and sighted participants (M = 3.57, SD = 0.97), (F = 5.37, p = 0.007). Only the difference between late blind and sighted was significant (p = 0.006).

The percentage of participants that reported they thought they would be able to recognize the smell of someone important was similar for early blind (80.00 %), late blind, (73.33 %), and sighted participants (73.33 %), ($\chi 2$ = 0.72, $p > 0.05$).

The number of odors attended to was higher for late blind (M = 10.87, SD = 9.46) than for early blind (M = 5.13, SD = 3.74) and sighted participants (M = 4.63, SD = 4.10), (F = 6.13, p = 0.004). Only the differences between late and early blind (p = 0.025) and between late blind and sighted participants (p = 0.004) were significant.

Imagery ability was analyzed with nonparametric Kruskal-Wallis tests. The results indicated a significant difference in olfactory imagery ratings between early blind (median = 4.06), late blind (median = 3.88), and sighted participants (median = 3.40), ($\chi 2$ = 6.53, p =

0.038). Follow-up Mann-Withney U tests indicated that early and late blind participants had significantly higher ratings than the sighted (Z = -2.05, p = 0.04 and Z = -2.10, p = 0.036, respectively). Similarly, the auditory imagery ratings differed significantly between early blind (median = 4.75), late blind (median = 4.63), and sighted participants (median = 4.25), ($\chi 2$ = 8.65, p = 0.013). Mann-Withney U tests indicated that only the difference between early blind and sighted participants was significant (Z = -2.70, p = 0.007).
