# Supplementary material for: From Perception to Metacognition: Auditory and Olfactory Functions in Early Blind, Late Blind, and Sighted Individuals
Source: Front Psychol. 2016 Sep 27;7:1450. doi: 10.3389/fpsyg.2016.01450 (PMC5037222; doi:10.3389/fpsyg.2016.01450)
Supplement: Supplementary file 5 [file Image3.pdf]

Supplementary Material

From perception to metacognition: Auditory and olfactory functions in early blind, late blind, and sighted individuals

Stina Cornell Kärnekull[1†], Artin Arshamian[1,2,3†*] Mats E Nilsson[1], Maria Larsson[1]
*** Correspondence:** Dr. Artin Arshamian: artin.arshamian@ki.se

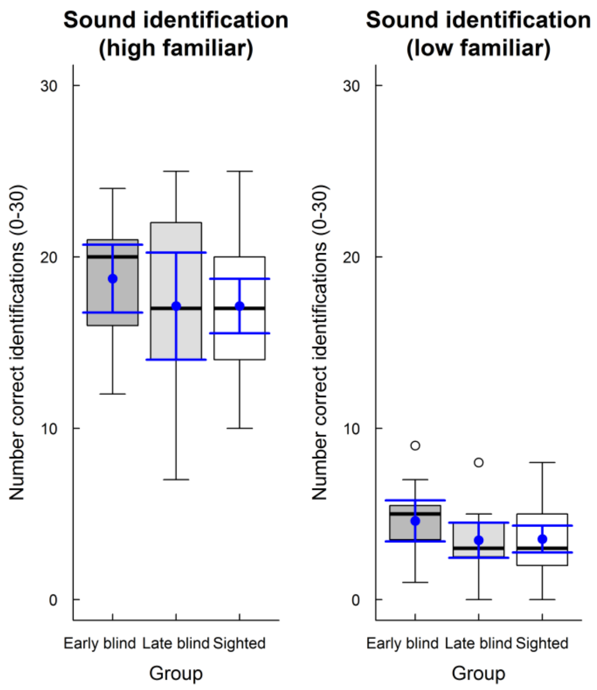

Fig. S3. Boxplots of identification for high and low familiar sounds, separately for early blind (dark grey boxes), late blind (light grey boxes), and sighted (white boxes) participants. The boxes indicate the 25th, 50th (median), and 75th percentiles of the distribution (lower, middle, and upper horizontal lines of the box). The upper hinges indicate the maximum value of the variable located within a distance of 1.5 times the inter-quartile range above the 75th percentile. The lower hinges indicate the corresponding distance to the 25th percentile value. Circles indicate values outside these hinges (outliers). The means and 95 % confidence intervals (dots and error bars in blue) are superimposed on the boxplots.
